# Supplementary material for: Gene expression data support the hypothesis that Isoetes rootlets are true roots and not modified leaves
Source: Sci Rep. 2020 Dec 9;10:21547. doi: 10.1038/s41598-020-78171-y (PMC7725790; doi:10.1038/s41598-020-78171-y)
Supplement: Supplementary file 1 — Supplementary Legends. [file 41598_2020_78171_MOESM1_ESM.docx]

Supplementary Figure S1. Comparison of root and leaf transcriptomes of *Arabidopsis thaliana*, *I. echinospora* and *Selaginella moellendorffii* based on 1690 non photosynthetic orthologs. Comparison of gene expression profiles by PCoA in the transcriptomes of *A. thaliana,* circles, *S. moellendorffii,* squares and *I. echinospora,* triangles. Leaf samples coloured green, root samples orange. A, comparison of principal coordinate axis 1 and 2. B, comparison of principal coordinates axis 1 and 3. C, comparison of principal coordinate axis 2 and 3. Values in brackets on each axis describe the percentage of total variance accounted for by each axis. Axis 1 separates gene expression in the three species. Axis 2 distinguishes gene expression between the two lycophytes transcriptomes, *I. echinospora* and *S. moellendorffii* from *A. thaliana.* Axis 3 distinguishes between the leaf samples and the root samples in each transcriptome.

Supplementary Figure S2. There are no RSL genes in the *Salvinia cucullata* genome or proteome. Gene tree analysis of RSL and related basic Helix-Loop-Helix (bHLH) transcription factors in a subset of land plant species. There are not *S. cucullata* genes in the RSL clade instead closely related *S. cucullate* genes are members of subfamily XI. Maximum likelihood gene tree of bHLH transcription factors generated in PhyML 3.0. Gene names: black, bryophytes; green, lycopsids; orange, ferns; blue, angiosperms. The RSL genes are grouped into two monophyletic classes; Class I and Class II. Species name abbreviations: Phpat, *Physcomitrella patens*; Mapoly, *Marchantia polymorpha*; Some, *Selaginella moellendorffii*; Sk, *Selaginella kraussiana*; Ie_Transcript, *Isoetes echinospora*; Sacu, *Salvinia cucullata*; Azfi, *Azolla filiculoides*; AmTr, *Amborella trichopoda*; AT *Arabidopsis thaliana*. Branch support as Shimodaira-Hasegawa-like approximate likelihood ratio tests.
