# Supplementary figures and images for: Gene expression data support the hypothesis that Isoetes rootlets are true roots and not modified leaves
Source: Sci Rep. 2020 Dec 9;10:21547. doi: 10.1038/s41598-020-78171-y (PMC7725790; doi:10.1038/s41598-020-78171-y)

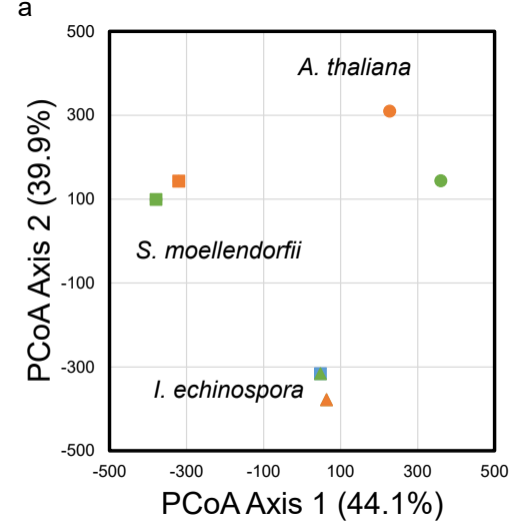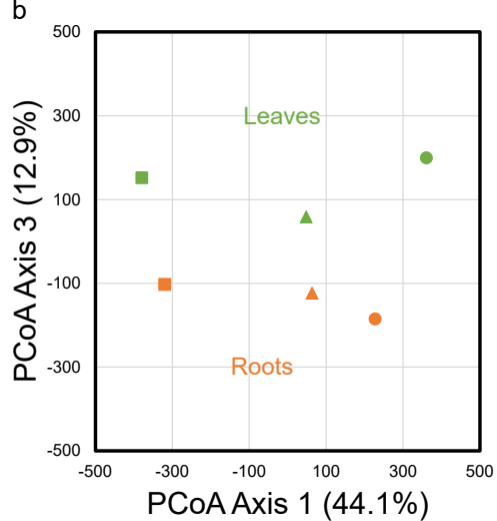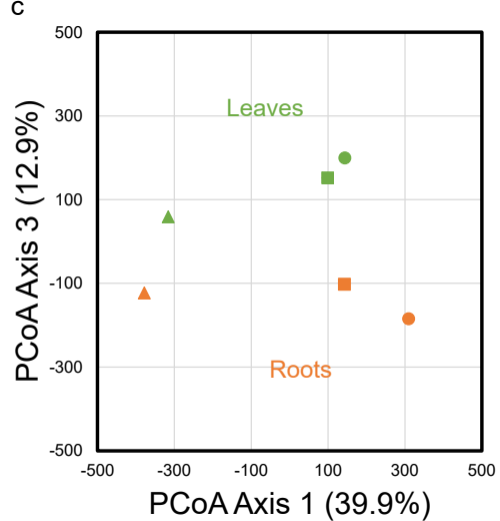

Supplement: Supplementary file 2 — Supplementary Figure S1. [file 41598_2020_78171_MOESM2_ESM.pdf]

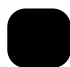

Bryophytes

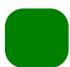

Lycophytes

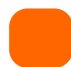

Ferns

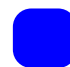

Angiosperms

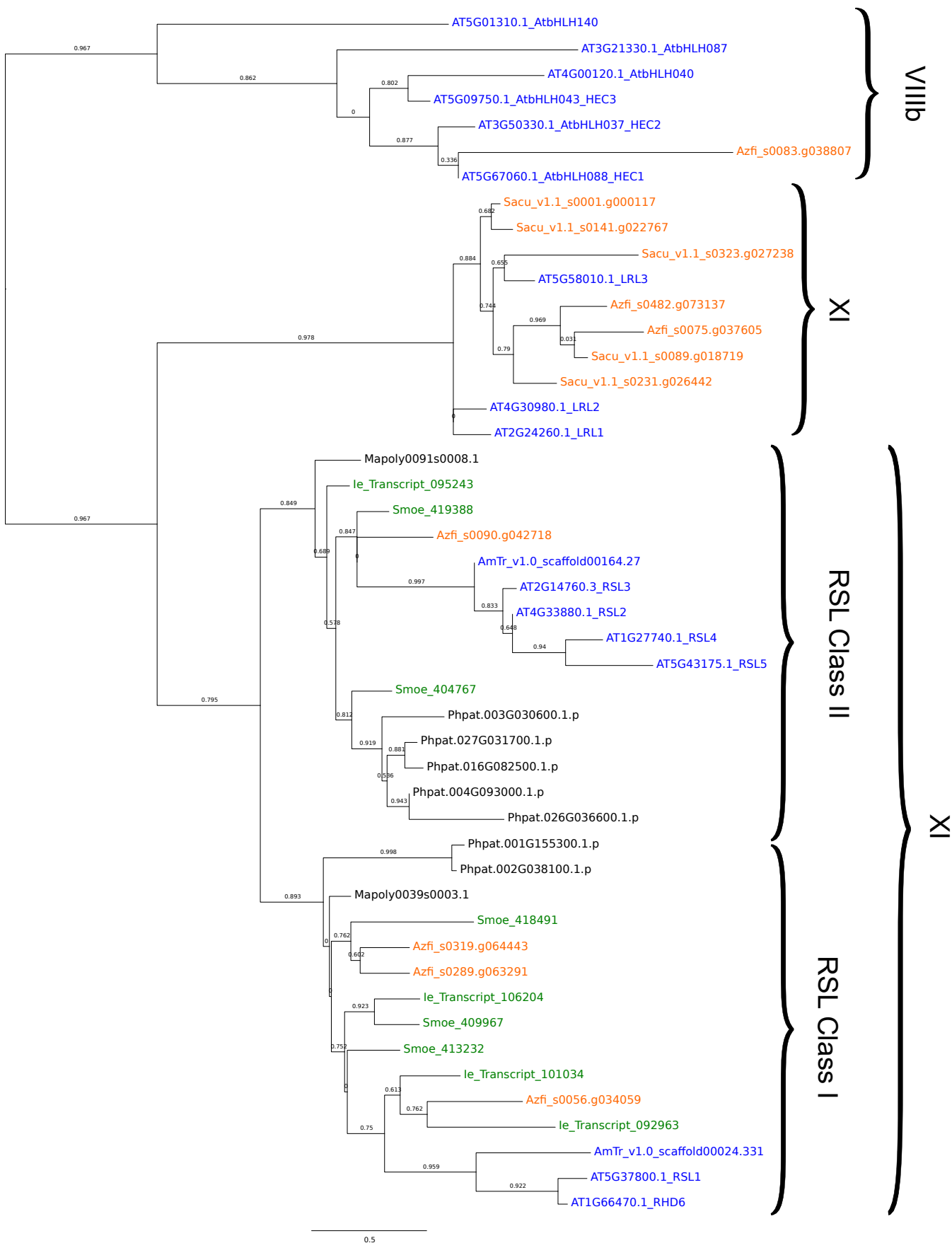

Supplement: Supplementary file 3 — Supplementary Figure S2. [file 41598_2020_78171_MOESM3_ESM.pdf]
